# Supplementary material for: NME4 mediates metabolic reprogramming and promotes nonalcoholic fatty liver disease progression
Source: EMBO Rep. 2023 Dec 14;25(1):22. doi: 10.1038/s44319-023-00012-6 (PMC10897415; doi:10.1038/s44319-023-00012-6)
Supplement: Supplementary file 1 — Appendix Figures and Legends [file 44319_2023_12_MOESM1_ESM.pdf]

Appendix for

NME4 mediates metabolic reprogramming and promotes non-alcoholic fatty liver disease progression

Shaofang Xie, Lei Yuan, Yue Sui, Shan Feng, Hengle Li, Xu Li

Index

Appendix Figure S1 Page 2

Appendix Figure S2 Page 3

Appendix Figure S3 Page 4

Appendix Figure S4 Page 5

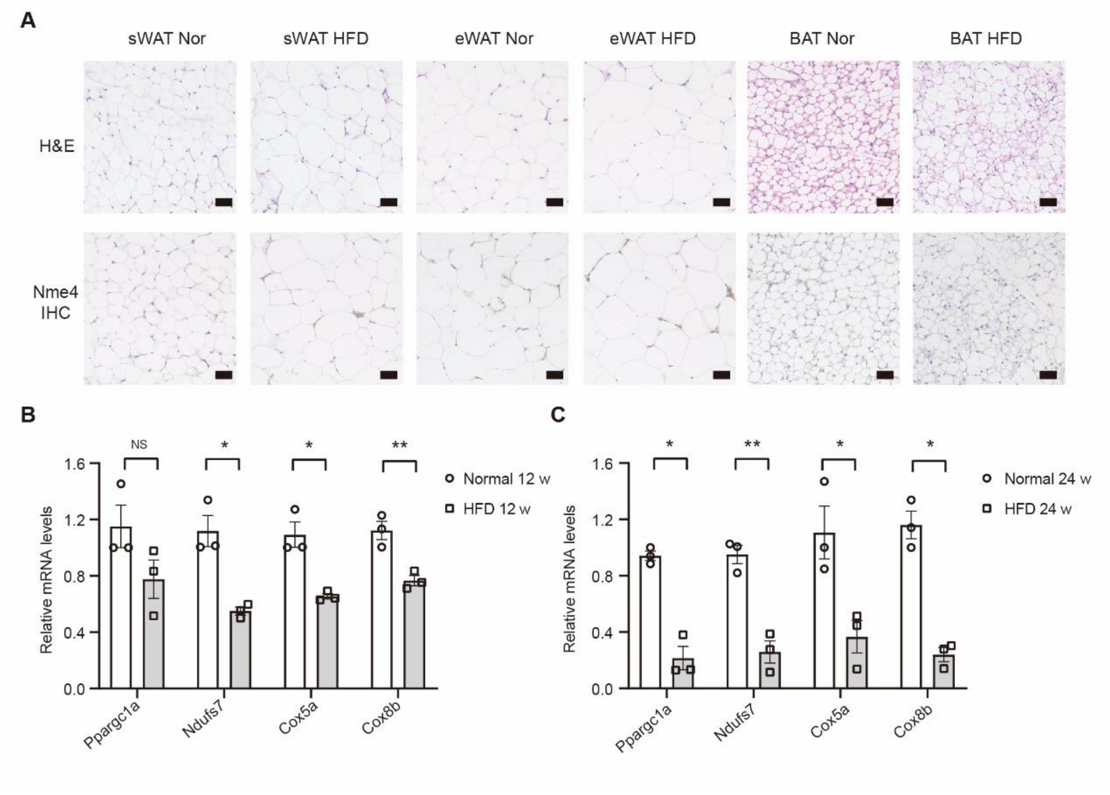

Appendix Figure S1. NME4 expression in adipose tissue and mitochondrial function gene expression in liver tissue.

A. H&E staining and IHC staining of sWAT, eWAT and BAT tissue fed with HFD 12 weeks were quantified by Image-Pro Plus (IPP). Scale bars, 50 μm**.**

B, C. Relative mRNA levels of mitochondrial functional gene in the livers of the mice fed a normal diet or HFD for 12 weeks and 24 weeks were measured by RT–qPCR. The mRNA level was detected by qPCR and normalized to β-actin. Biological replicates, n = 3, unpaired Student's t-test; *P values < 0.05, **P values < 0.01, NS-P values > 0.05. Data are shown as the Mean ± S.E.M.

Data information: sWAT, subcutaneous white adipose tissue; eWAT, epididymal white adipose tissue; BAT, brown adipose tissue; Nor, normal diet; HFD, High fat diet; w, week.

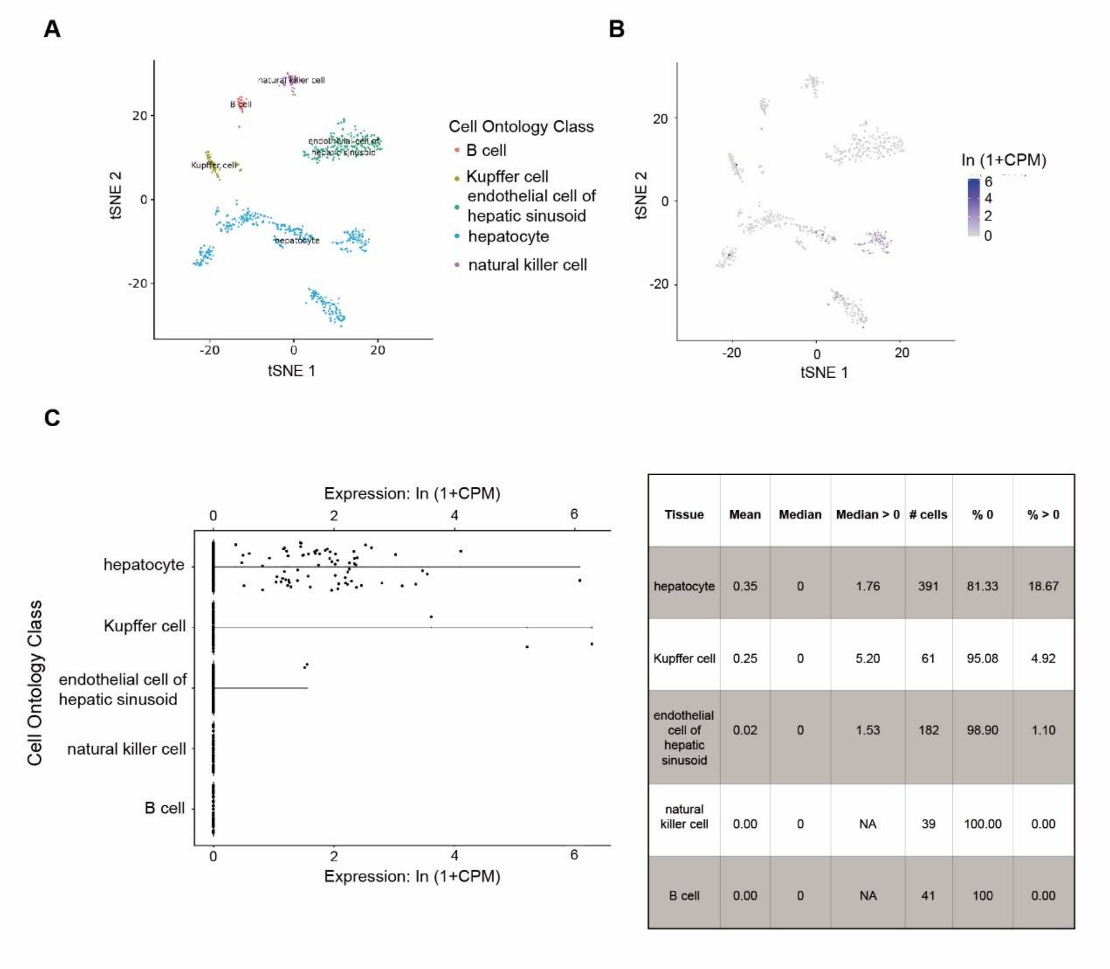

Tissue	Mean	Median	Median > 0	# cells	% 0	% > 0
hepatocyte	0.35	0	1.76	391	81.33	18.67
Kupffer cell	0.25	0	5.20	61	95.08	4.92
endothelial cell of hepatic sinusoid	0.02	0	1.53	182	98.90	1.10
natural killer cell	0.00	0	NA	39	100.00	0.00
B cell	0.00	0	NA	41	100	0.00

Appendix Figure S2. Single cell dataset indicated Nme4 mainly expresses in hepatocytes

A. t-SNE plot of all cells collected by FACS of mouse liver tissue, colored by cell type.

B. Normalized Nme4 gene expression level in different cell types from liver tissue (purple: high, white: low).

C. Violin plots (left) indicating Nme4 gene expression level and distributions of selected genes within cell clusters corresponding to panel and tabular statistical analysis (right) of violin plots.

Data information: CPM, Counts per million.

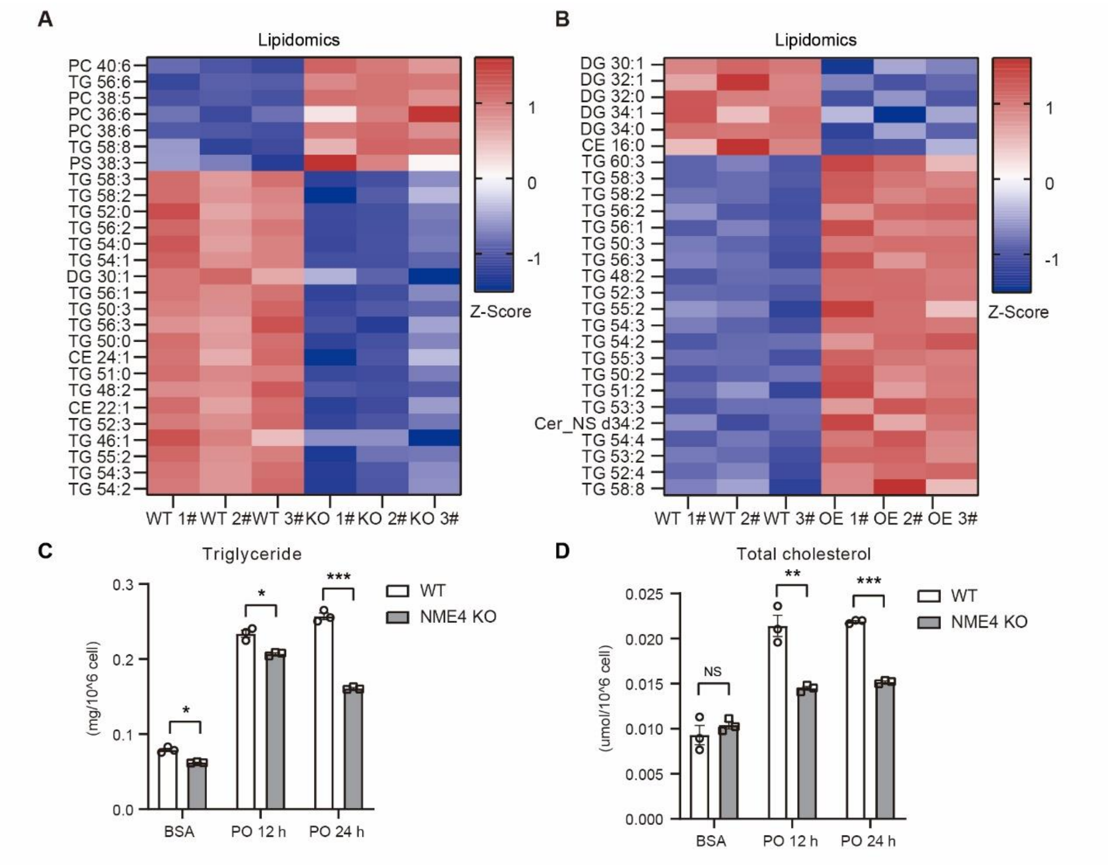

Appendix Figure S3. NME4 promotes lipid accumulation by increasing triglyceride levels in human liver cells.

A, B. Heatmap of lipids whose levels were significantly changed in wild-type and NME4-KO Bel-7402 cells (A) and wild-type and NME4-OE SK-Hep1 cells (B) are shown.

C, D. Bel-7402 cells were treated with PO for 12 and 24 h. Triglyceride (C) and total cholesterol (D) levels were measured. Biological replicates, n = 3, unpaired Student's t-test; *P values < 0.05, **P values < 0.01, ***P values < 0.001, NS-P values > 0.05. Data are shown as the Mean ± S.E.M.

Data information: KO, knockout; OE, overexpression; h, hour.

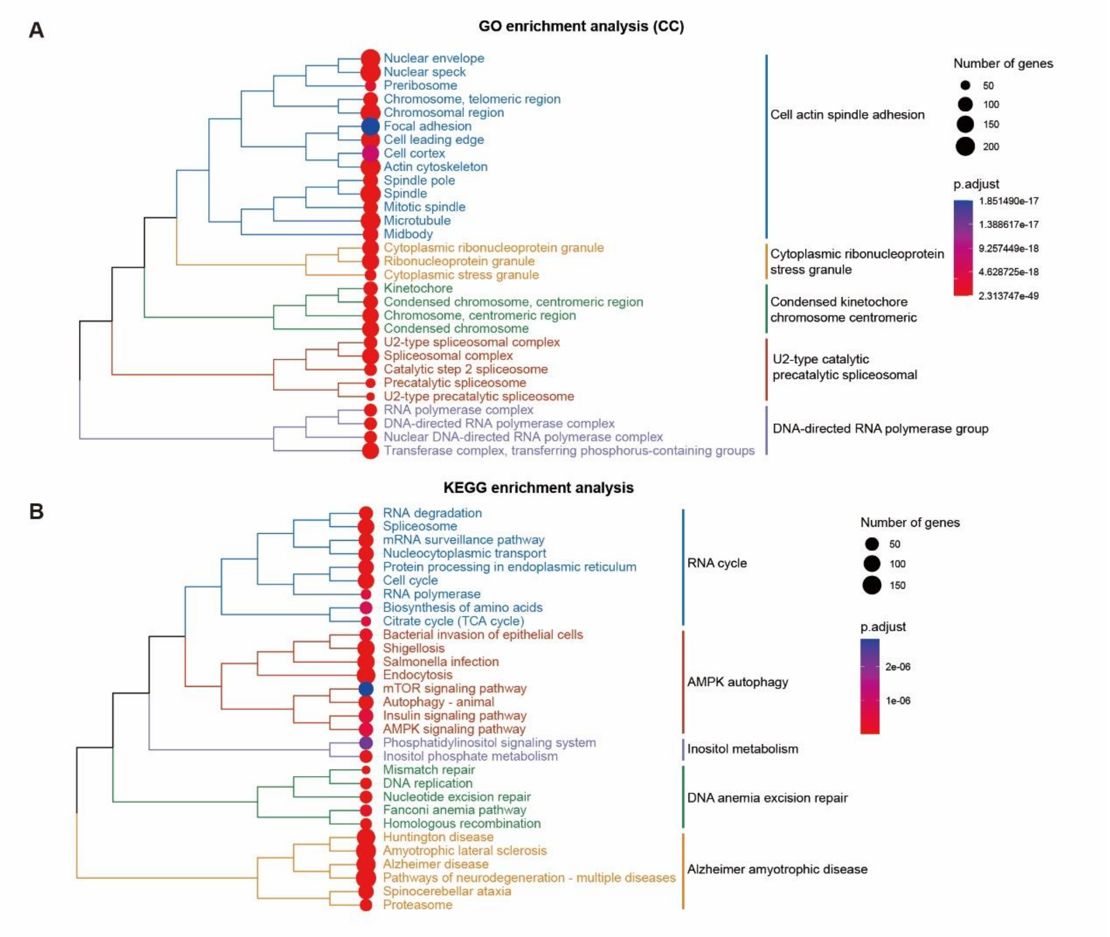

Appendix Figure S4. The GO and KEGG enrichment of mNME4 HCIPs

A, B TurboID-MS mNME4 HCIPs were enriched by Gene Ontology analysis in Cellular Components (CC) (A) and by KEGG analysis (B) which were analyzed in Hitplot. Here only showed the top 5 pathway. P.adjust $\leqslant 0.05$ was cut off which was adjusted by Benjamini and Hochberg FDR (BH).
