# Supplementary figures and images for: NME4 mediates metabolic reprogramming and promotes nonalcoholic fatty liver disease progression
Source: EMBO Rep. 2023 Dec 14;25(1):22. doi: 10.1038/s44319-023-00012-6 (PMC10897415; doi:10.1038/s44319-023-00012-6)

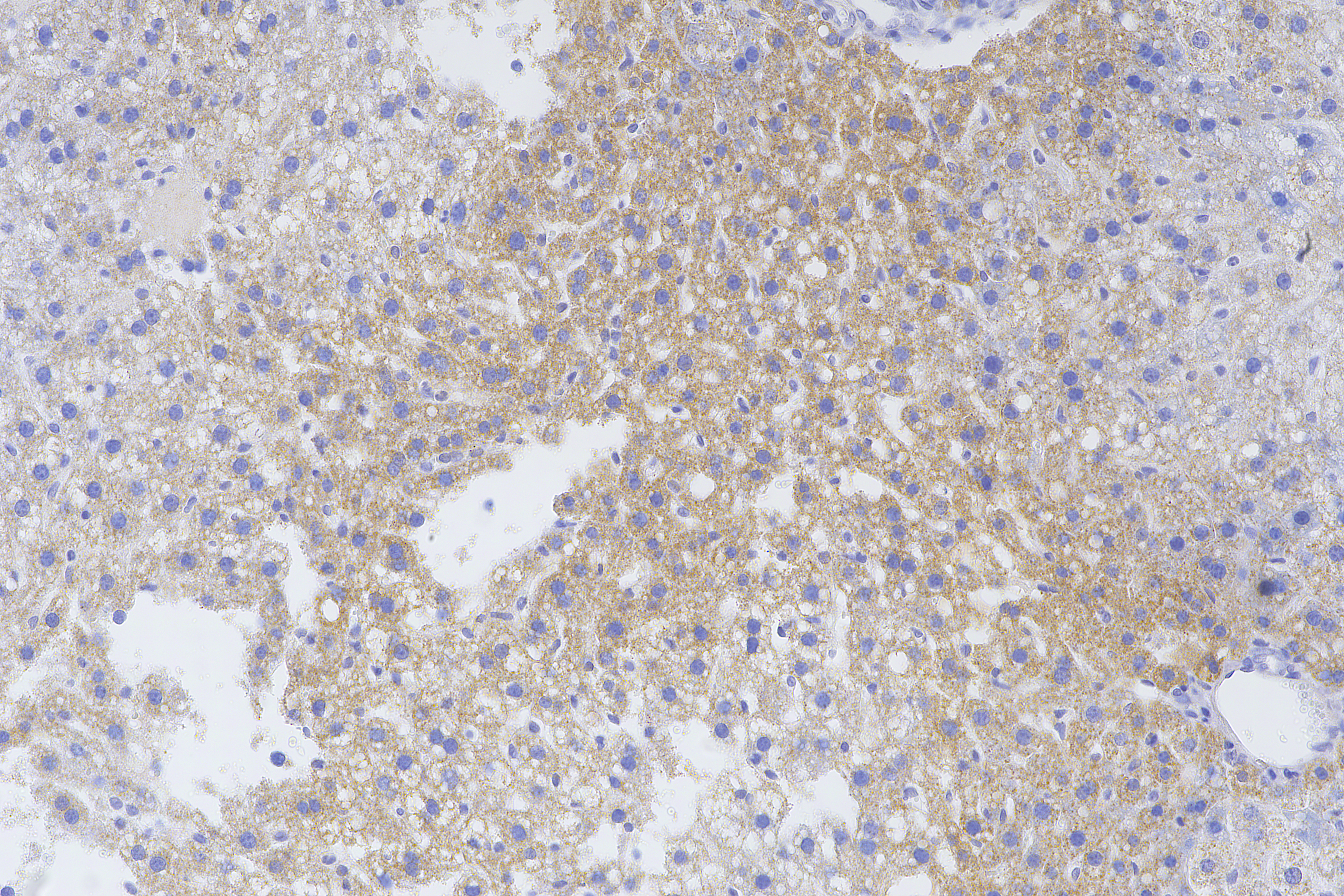

Supplement: Supplementary file 14 — Source Data Fig. 1 [file 44319_2023_12_MOESM14_ESM.zip › Source Data Figure 1/1G/HFD 6W IHC staining.tif]

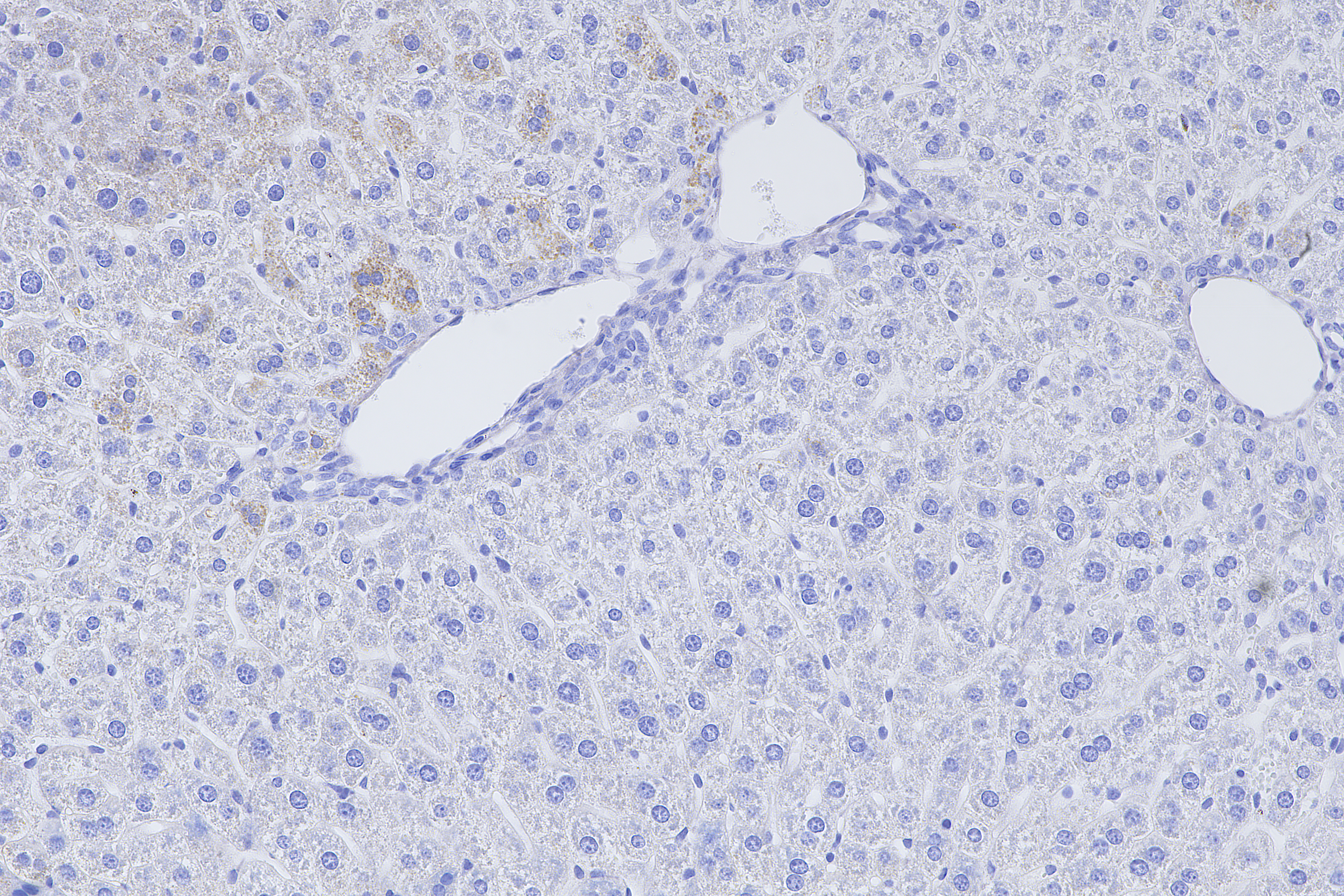

Supplement: Supplementary file 14 — Source Data Fig. 1 [file 44319_2023_12_MOESM14_ESM.zip › Source Data Figure 1/1G/NOR 6W IHC staining.tif]

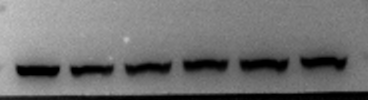

Supplement: Supplementary file 14 — Source Data Fig. 1 [file 44319_2023_12_MOESM14_ESM.zip › Source Data Figure 1/1I/Actin 18W.tif]

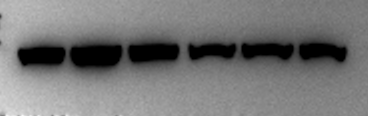

Supplement: Supplementary file 14 — Source Data Fig. 1 [file 44319_2023_12_MOESM14_ESM.zip › Source Data Figure 1/1I/Actin 24W.tif]

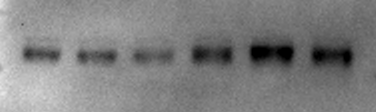

Supplement: Supplementary file 14 — Source Data Fig. 1 [file 44319_2023_12_MOESM14_ESM.zip › Source Data Figure 1/1I/NME4 18W.tif]

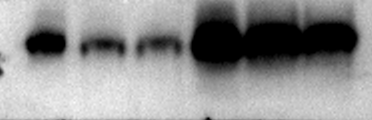

Supplement: Supplementary file 14 — Source Data Fig. 1 [file 44319_2023_12_MOESM14_ESM.zip › Source Data Figure 1/1I/NME4 24W.Tif]

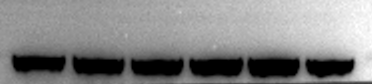

Supplement: Supplementary file 14 — Source Data Fig. 1 [file 44319_2023_12_MOESM14_ESM.zip › Source Data Figure 1/1L/Actin.tif]

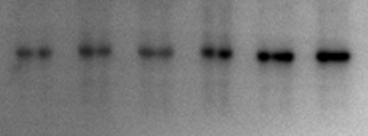

Supplement: Supplementary file 14 — Source Data Fig. 1 [file 44319_2023_12_MOESM14_ESM.zip › Source Data Figure 1/1L/NME4.tif]

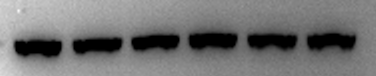

Supplement: Supplementary file 14 — Source Data Fig. 1 [file 44319_2023_12_MOESM14_ESM.zip › Source Data Figure 1/1M/Actin.tif]

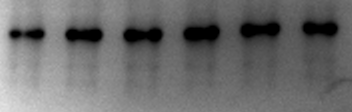

Supplement: Supplementary file 14 — Source Data Fig. 1 [file 44319_2023_12_MOESM14_ESM.zip › Source Data Figure 1/1M/NME4.tif]

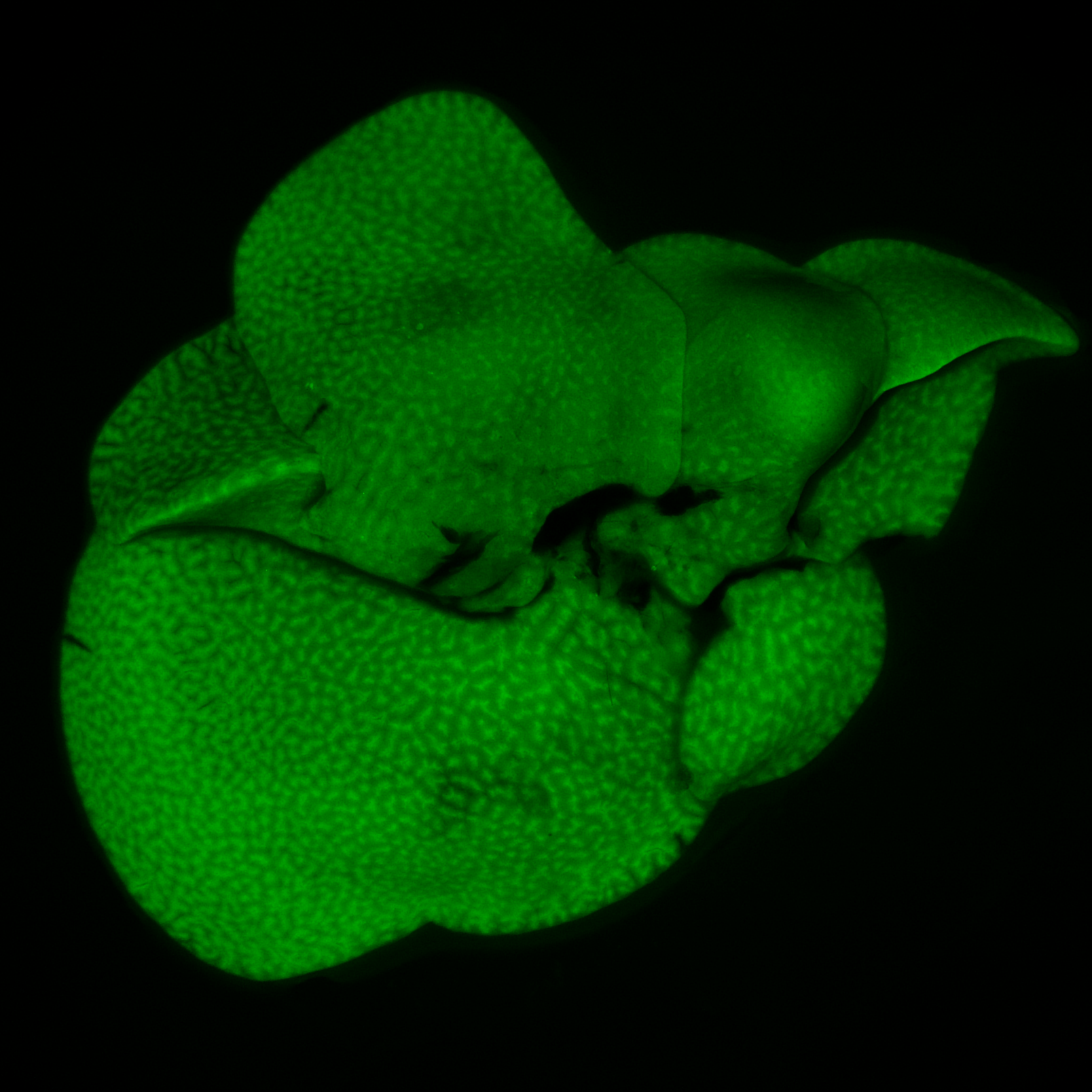

Supplement: Supplementary file 16 — Source Data Fig. 3 [file 44319_2023_12_MOESM16_ESM.zip › Source Data Figure 3/3 A/shNme4.tif]

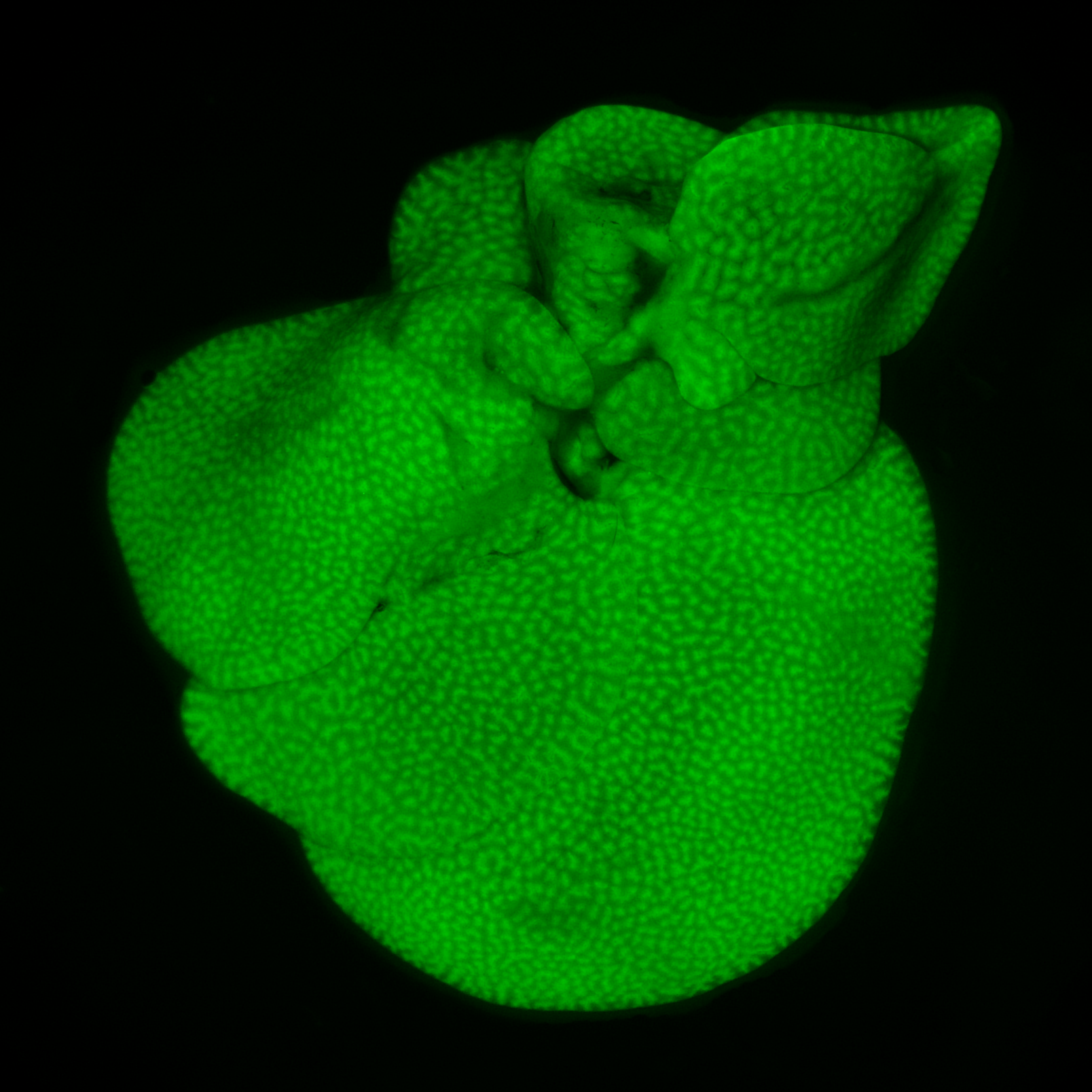

Supplement: Supplementary file 16 — Source Data Fig. 3 [file 44319_2023_12_MOESM16_ESM.zip › Source Data Figure 3/3 A/shSm.tif]

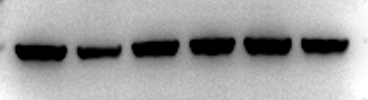

Supplement: Supplementary file 16 — Source Data Fig. 3 [file 44319_2023_12_MOESM16_ESM.zip › Source Data Figure 3/3 K/Actin.tif]

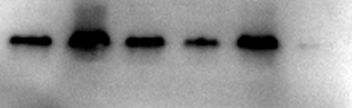

Supplement: Supplementary file 16 — Source Data Fig. 3 [file 44319_2023_12_MOESM16_ESM.zip › Source Data Figure 3/3 K/NME4.tif]

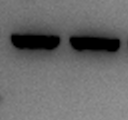

Supplement: Supplementary file 16 — Source Data Fig. 3 [file 44319_2023_12_MOESM16_ESM.zip › Source Data Figure 3/3 L/1 actin 2s.tif-1.tif]

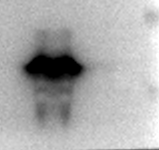

Supplement: Supplementary file 16 — Source Data Fig. 3 [file 44319_2023_12_MOESM16_ESM.zip › Source Data Figure 3/3 L/2 nme4 mouse 3s.tif-1.tif]

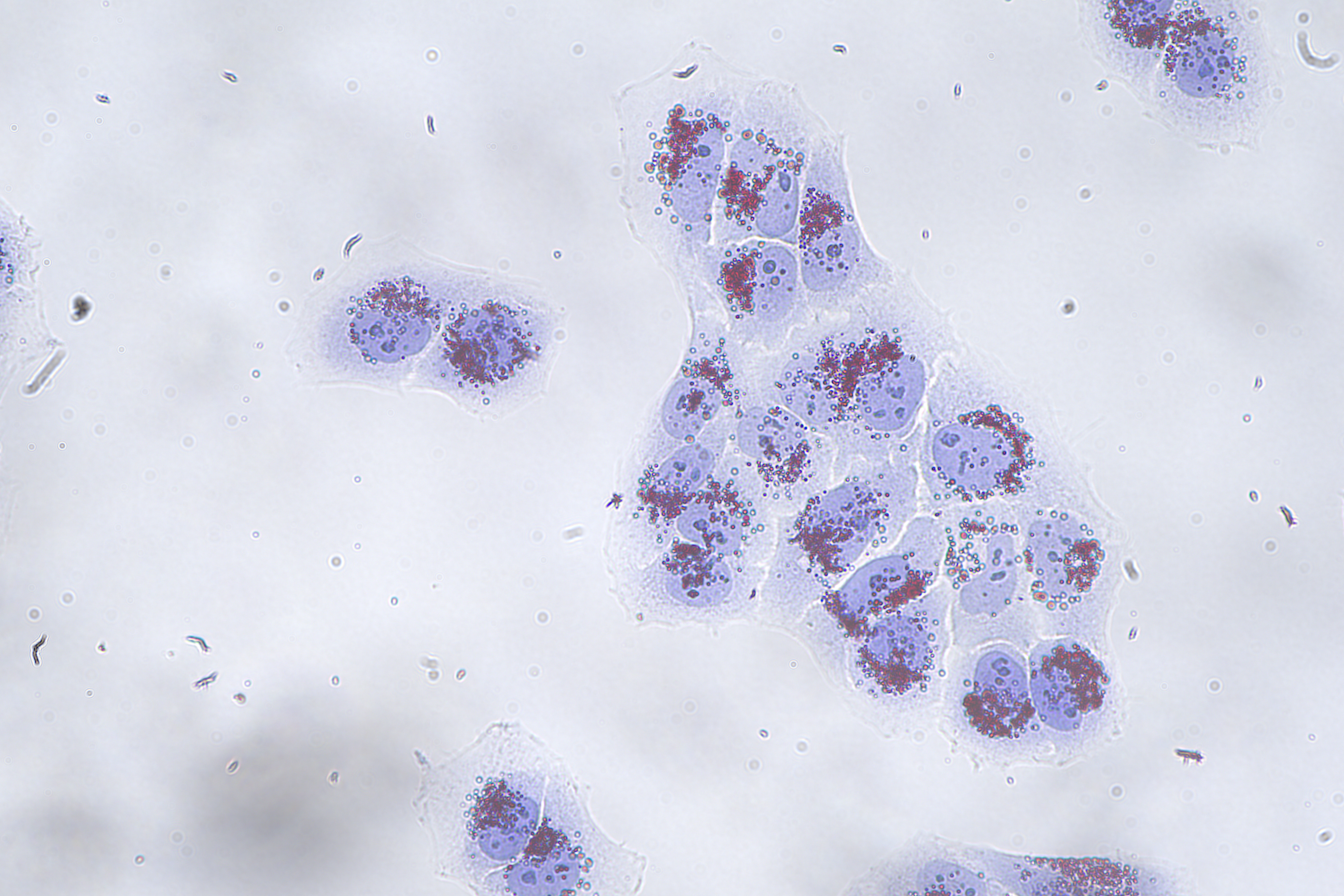

Supplement: Supplementary file 16 — Source Data Fig. 3 [file 44319_2023_12_MOESM16_ESM.zip › Source Data Figure 3/3 P/NME4 KO 12H.tif]

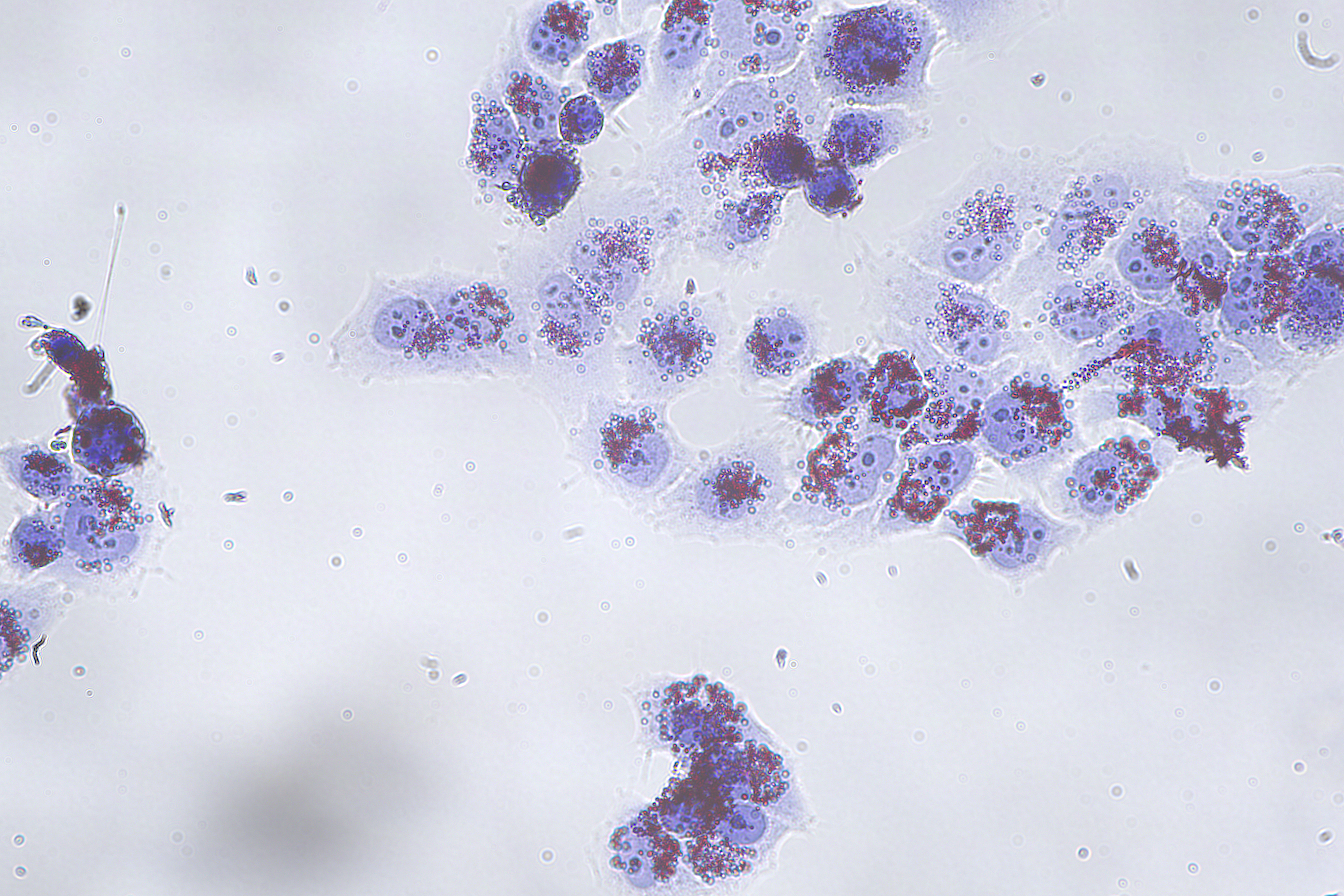

Supplement: Supplementary file 16 — Source Data Fig. 3 [file 44319_2023_12_MOESM16_ESM.zip › Source Data Figure 3/3 P/WT PO 12H.tif]

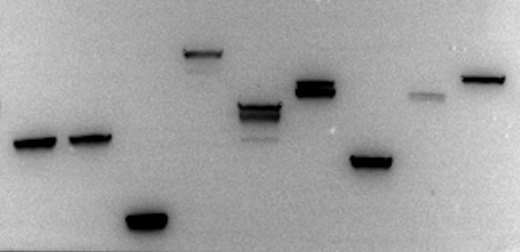

Supplement: Supplementary file 18 — Source Data Fig. 6 [file 44319_2023_12_MOESM18_ESM.zip › Source Data Figure 6/6 B/Flag Input.tif]

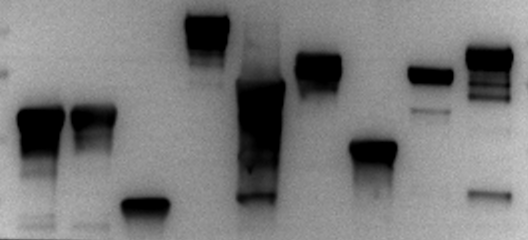

Supplement: Supplementary file 18 — Source Data Fig. 6 [file 44319_2023_12_MOESM18_ESM.zip › Source Data Figure 6/6 B/Flag IP.tif]

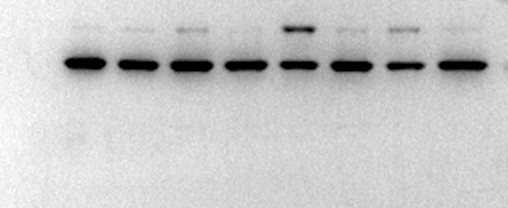

Supplement: Supplementary file 18 — Source Data Fig. 6 [file 44319_2023_12_MOESM18_ESM.zip › Source Data Figure 6/6 B/Myc Input .Tif]

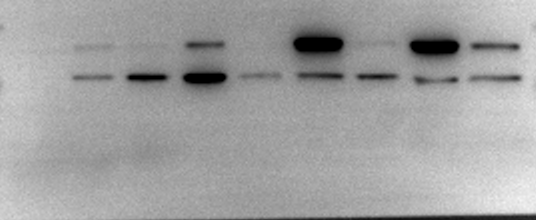

Supplement: Supplementary file 18 — Source Data Fig. 6 [file 44319_2023_12_MOESM18_ESM.zip › Source Data Figure 6/6 B/Myc IP.tif]

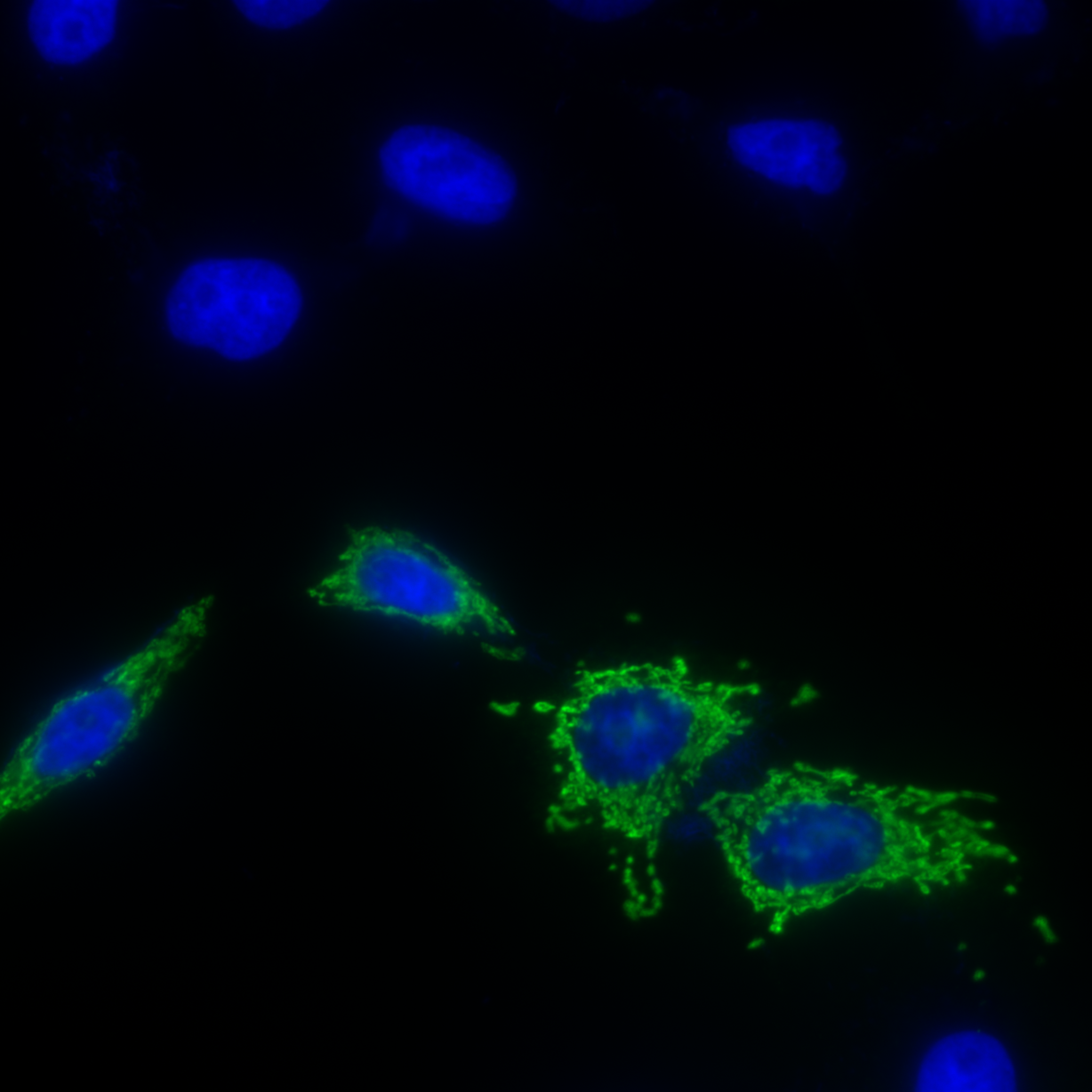

Supplement: Supplementary file 19 — Source Data Fig. 7 [file 44319_2023_12_MOESM19_ESM.zip › Source Data Figure 7/7 A/NME4 FL.tif]

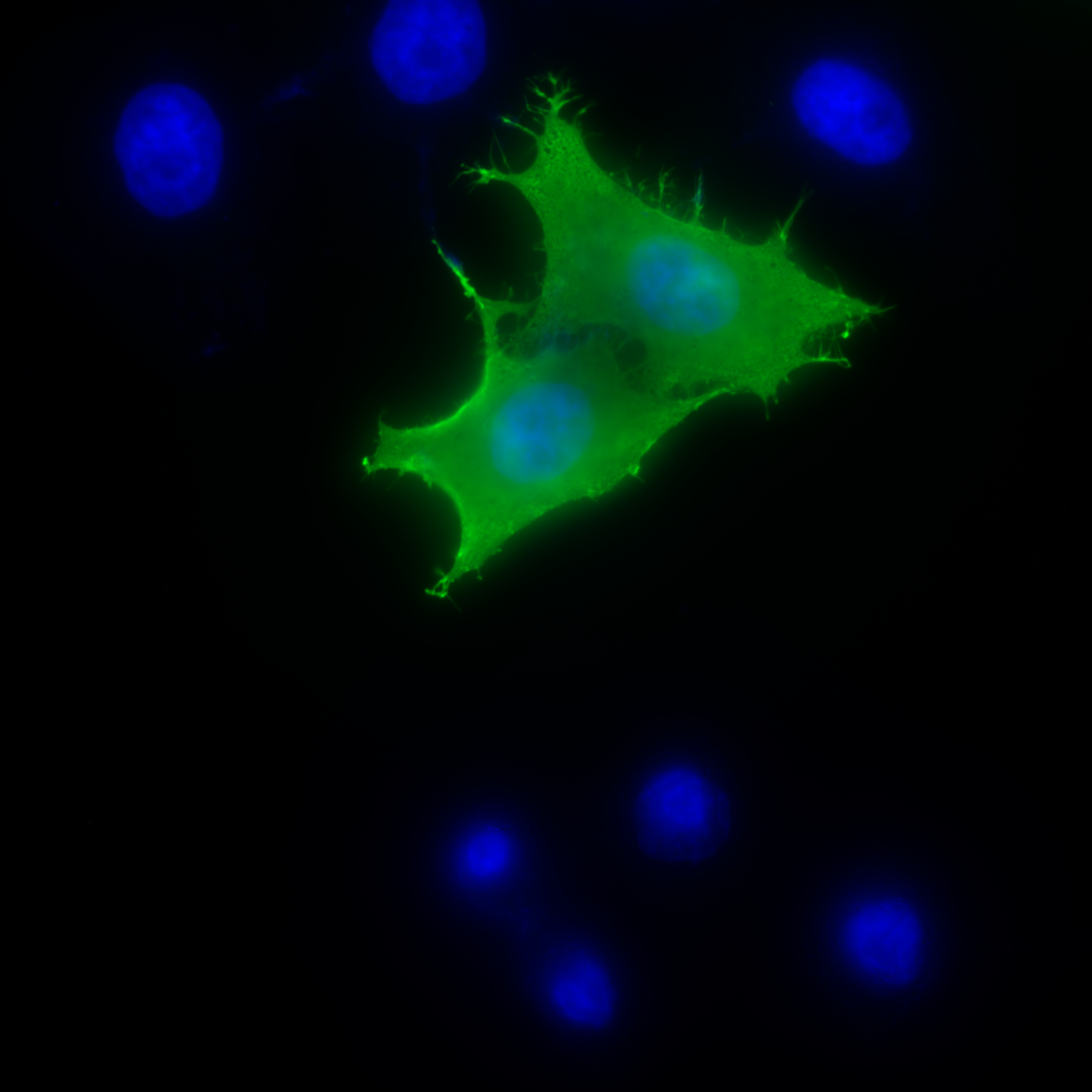

Supplement: Supplementary file 19 — Source Data Fig. 7 [file 44319_2023_12_MOESM19_ESM.zip › Source Data Figure 7/7 A/NME4 Δmito.tif]

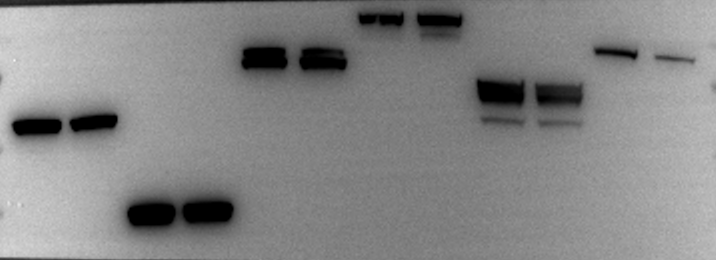

Supplement: Supplementary file 19 — Source Data Fig. 7 [file 44319_2023_12_MOESM19_ESM.zip › Source Data Figure 7/7 B/Flag Input.tif]

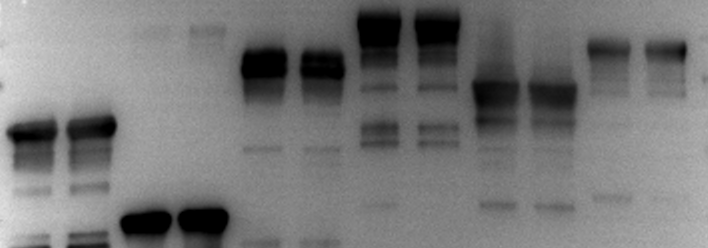

Supplement: Supplementary file 19 — Source Data Fig. 7 [file 44319_2023_12_MOESM19_ESM.zip › Source Data Figure 7/7 B/Flag IP.tif]

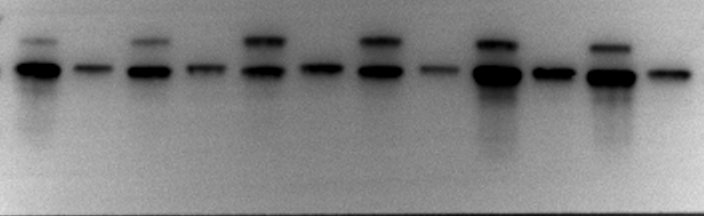

Supplement: Supplementary file 19 — Source Data Fig. 7 [file 44319_2023_12_MOESM19_ESM.zip › Source Data Figure 7/7 B/Myc Input.tif]

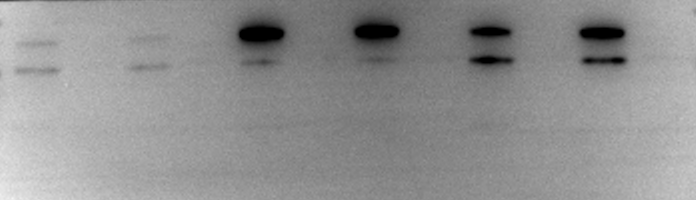

Supplement: Supplementary file 19 — Source Data Fig. 7 [file 44319_2023_12_MOESM19_ESM.zip › Source Data Figure 7/7 B/Myc IP.tif]
