# Supplementary material for: NME4 mediates metabolic reprogramming and promotes nonalcoholic fatty liver disease progression
Source: EMBO Rep. 2023 Dec 14;25(1):22. doi: 10.1038/s44319-023-00012-6 (PMC10897415; doi:10.1038/s44319-023-00012-6)
Supplement: Supplementary file 20 — Figure Source Data External Links [file 44319_2023_12_MOESM20_ESM.docx]

1D, 3F, 3I, 3N, 3O, 6C, 7E are available in Biostudy, link as below:

[https://www.ebi.ac.uk/biostudies/studies/S-BSST1196](https://apc01.safelinks.protection.outlook.com/?url=https%3A%2F%2Fwww.ebi.ac.uk%2Fbiostudies%2Fstudies%2FS-BSST1196&data=05%7C01%7Clixu%40westlake.edu.cn%7C78dc308017b84f6d2c4108dbb904be67%7C7e82de2f7ef644169b9644c1457be81b%7C1%7C0%7C638307202258745462%7CUnknown%7CTWFpbGZsb3d8eyJWIjoiMC4wLjAwMDAiLCJQIjoiV2luMzIiLCJBTiI6Ik1haWwiLCJXVCI6Mn0%3D%7C3000%7C%7C%7C&sdata=np0n0XgtrTaseXLqDTrSy9ac5ZlyjI9OP16Qg3Y6KAA%3D&reserved=0)
