# Supplementary material for: NME4 mediates metabolic reprogramming and promotes nonalcoholic fatty liver disease progression
Source: EMBO Rep. 2023 Dec 14;25(1):22. doi: 10.1038/s44319-023-00012-6 (PMC10897415; doi:10.1038/s44319-023-00012-6)
Supplement: Supplementary file 21 — Expanded View Figures [file 44319_2023_12_MOESM21_ESM.pdf]

Expanded View Figures

Figure EV1. NME4 is upregulated in fatty liver, and its level is correlated with NAFLD progression.

(**A**) Heatmap showing the tissue specificity of 80 mitochondrial proteins that were highly expressed in liver tissue. (**B**,**C**) H&E staining and Oil Red O staining of liver sections of the mice fed a high-fat diet (HFD) for the indicated times. Scale bars, 50 μm. (**D**,**E**) Western blots probed with antibodies against NME4 and Actin in the livers of mice fed a normal diet or a high-fat diet (HFD) for the 2 weeks and 6 weeks. (**F**) Relative mRNA levels of NME4 in the livers of the mice fed a normal diet or HFD for 2 weeks was measured by RT–qPCR. Biological replicates, $n = 3$. (**G**,**H**) Western blots probed with antibodies against NME4, UCP1 and Actin in the subcutaneous white adipose tissue (sWAT), epididymal white adipose tissue (eWAT) and brown adipose tissue (BAT) of mice fed a normal diet or a high-fat diet (HFD) for the 6 weeks. (**I**–**K**) Western blots probed with antibodies against NME4, UCP1 and Actin in the sWAT, eWAT, BAT and liver of mice fed a normal diet or a high-fat diet (HFD) for the 24 weeks. Data information: (**F**) data are presented as mean ± SEM. **P values ≤ 0.01 (Student's t test, unpaired). w, week; HFD high-fat diet, sWAT subcutaneous white adipose tissue, eWAT epididymal white adipose tissue, BAT brown adipose tissue.

 © The Author(s)

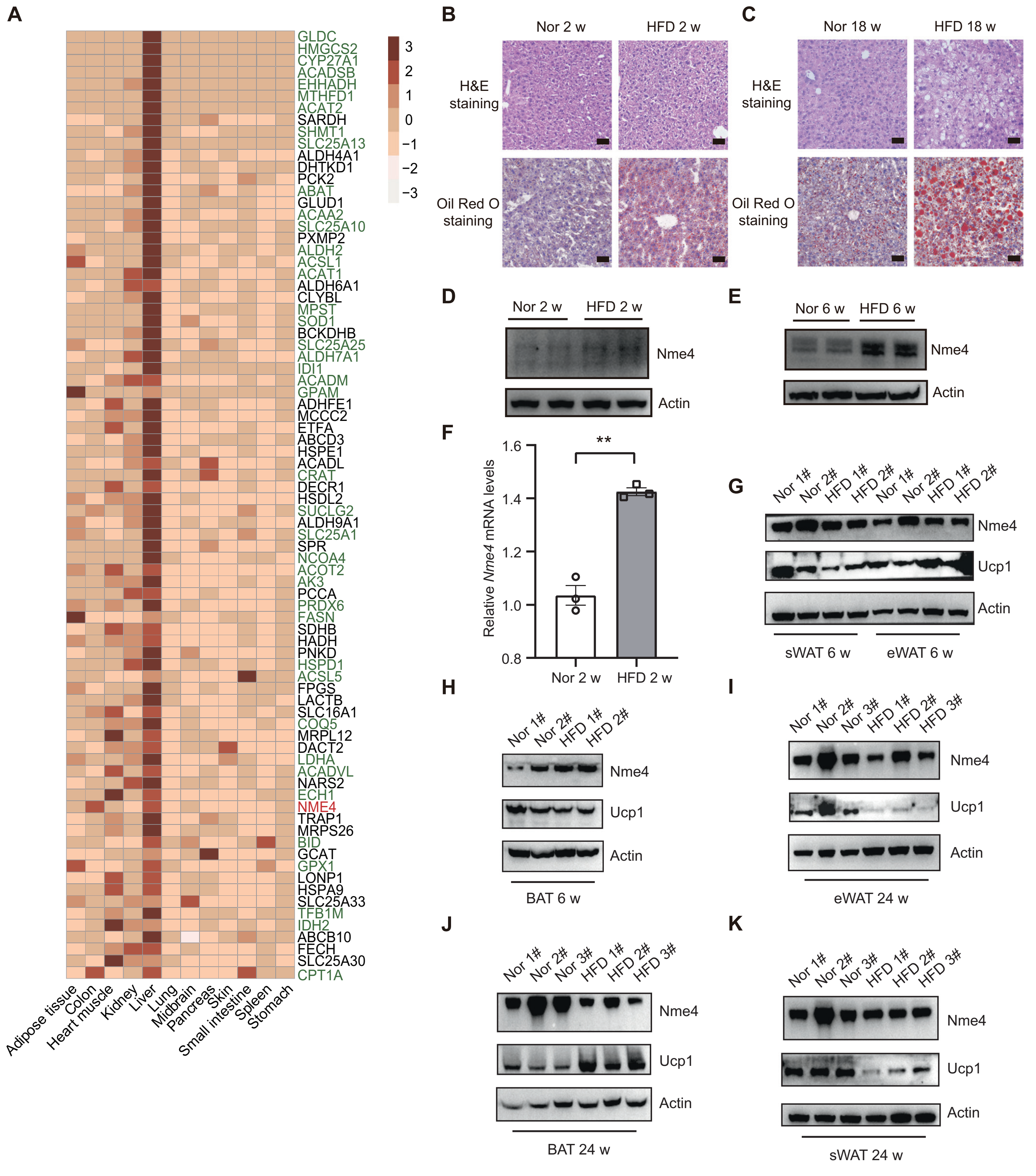
A
GLDC
HMGCS2
CYP27A1
ACADSB
EHHADH
MTHFD1
ACAT2
SARDH
SHMT1
SLC25A13
ALDH4A1
DHTKD1
PCK2
ABAT
GLUD1
ACAA2
SLC25A10
PXMP2
ALDH2
ACSL1
ACAT1
ALDH6A1
CLYBL
MPST
SOD1
BCKDHB
SLC25A25
ALDH7A1
IDI1
ACADM
GPAM
ADHFE1
MCCC2
ETFA
ABCD3
HSPE1
ACADL
CRAT
DECR1
HSDL2
SUCLG2
ALDH9A1
SLC25A1
SPR
NCOA4
ACOT2
AK3
PCCA
PRDX6
FASN
SDHB
HADH
PNKD
HSPD1
ACSL5
FPGS
LACTB
SLC16A1
COQ5
MRPL12
DACT2
LDHA
ACADVL
NARS2
ECH1
NME4
TRAP1
MRPS26
BID
GCAT
GPX1
LONP1
HSPA9
SLC25A33
TFB1M
IDH2
ABCB10
FECH
SLC25A30
CPT1A
3
2
1
0
−1
−2
−3
Adipose tissue
Colon
Heart muscle
Kidney
Liver
Lung
Midbrain
Pancreas
Skin
Small intestine
Spleen
Stomach
B
Nor 2 w
HFD 2 w
H&E staining
Oil Red O staining
C
Nor 18 w
HFD 18 w
H&E staining
Oil Red O staining
D
Nor 2 w
HFD 2 w
Nme4
Actin
E
Nor 6 w
HFD 6 w
Nme4
Actin
F
Relative Nme4 mRNA levels
1.6
1.4
1.2
1.0
0.8
**
Nor 2 w
HFD 2 w
G
Nor 1#
Nor 2#
HFD 1#
HFD 2#
Nor 1#
Nor 2#
HFD 1#
HFD 2#
Nme4
Ucp1
Actin
sWAT 6 w
eWAT 6 w
H
Nor 1#
Nor 2#
HFD 1#
HFD 2#
Nme4
Ucp1
Actin
BAT 6 w
I
Nor 1#
Nor 2#
Nor 3#
HFD 1#
HFD 2#
HFD 3#
Nme4
Ucp1
Actin
eWAT 24 w
J
Nor 1#
Nor 2#
Nor 3#
HFD 1#
HFD 2#
HFD 3#
Nme4
Ucp1
Actin
BAT 24 w
K
Nor 1#
Nor 2#
Nor 3#
HFD 1#
HFD 2#
HFD 3#
Nme4
Ucp1
Actin
sWAT 24 w

© The Author(s)

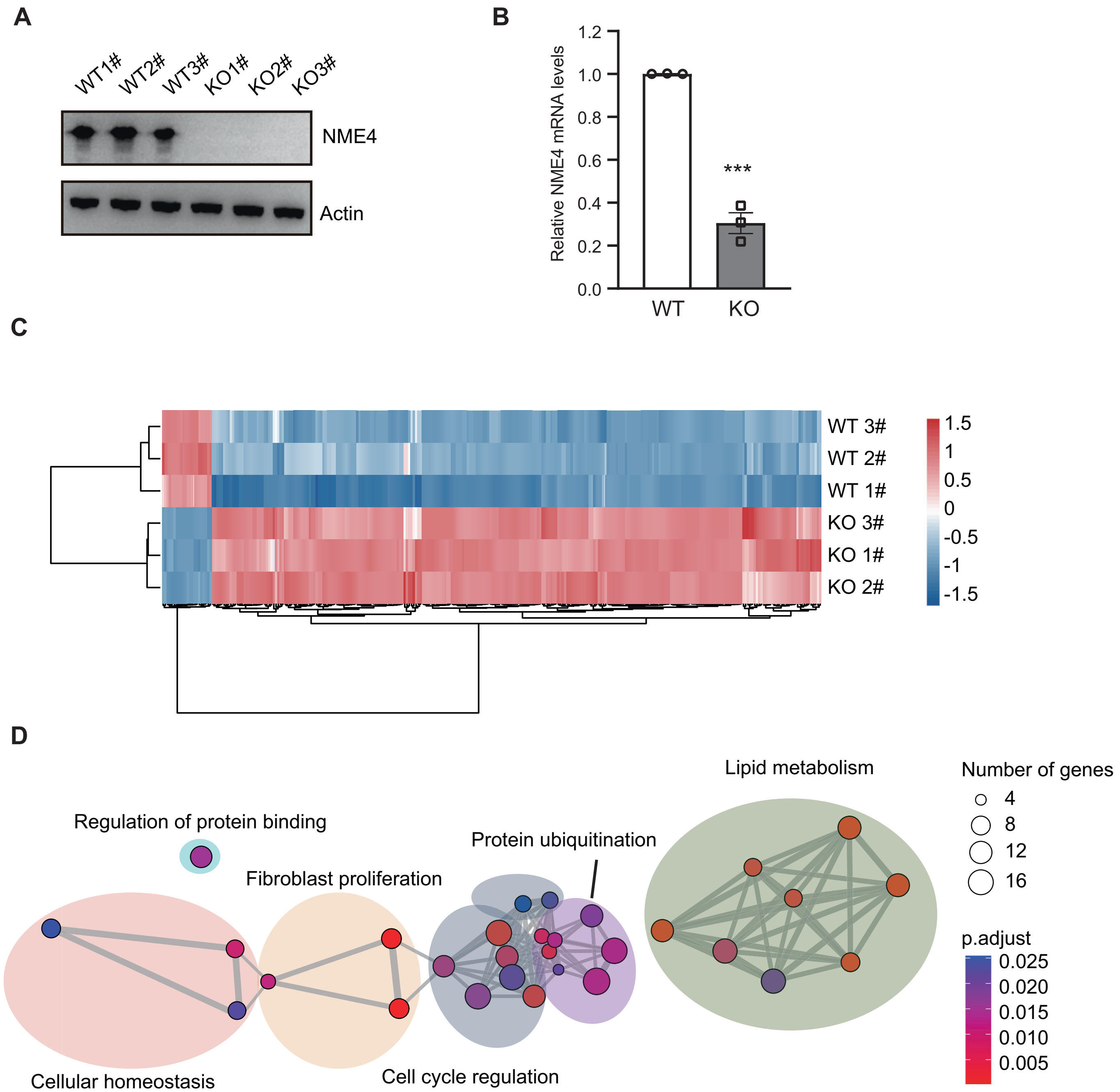

Figure EV2. Tandem mass tag-based quantitative proteomics reveal a correlation between NME4 and lipid metabolism.

(**A**) Representative western blotting (WB) analysis of NME4 expression in HEK293T stable cell lines. (**B**) Relative mRNA levels of NME4 in HEK293T WT and stable NME4 knockout cell lines. Biological replicates, $n = 3$. (**C**) Heatmap of proteins with significantly altered expression in TMT6-plex quantitative proteomics. (**D**) Pathway network enrichment of significantly upregulated proteins identified by TurboID-MS and performed using metascape database. *P*.adjust ≤ 0.05 was cut off which was adjusted by Benjamini and Hochberg FDR (BH). Data information: (**B**) data are presented as mean ± SEM. ****P* values ≤ 0.001 (Student's *t* test, unpaired). KO knockout.

 © The Author(s)

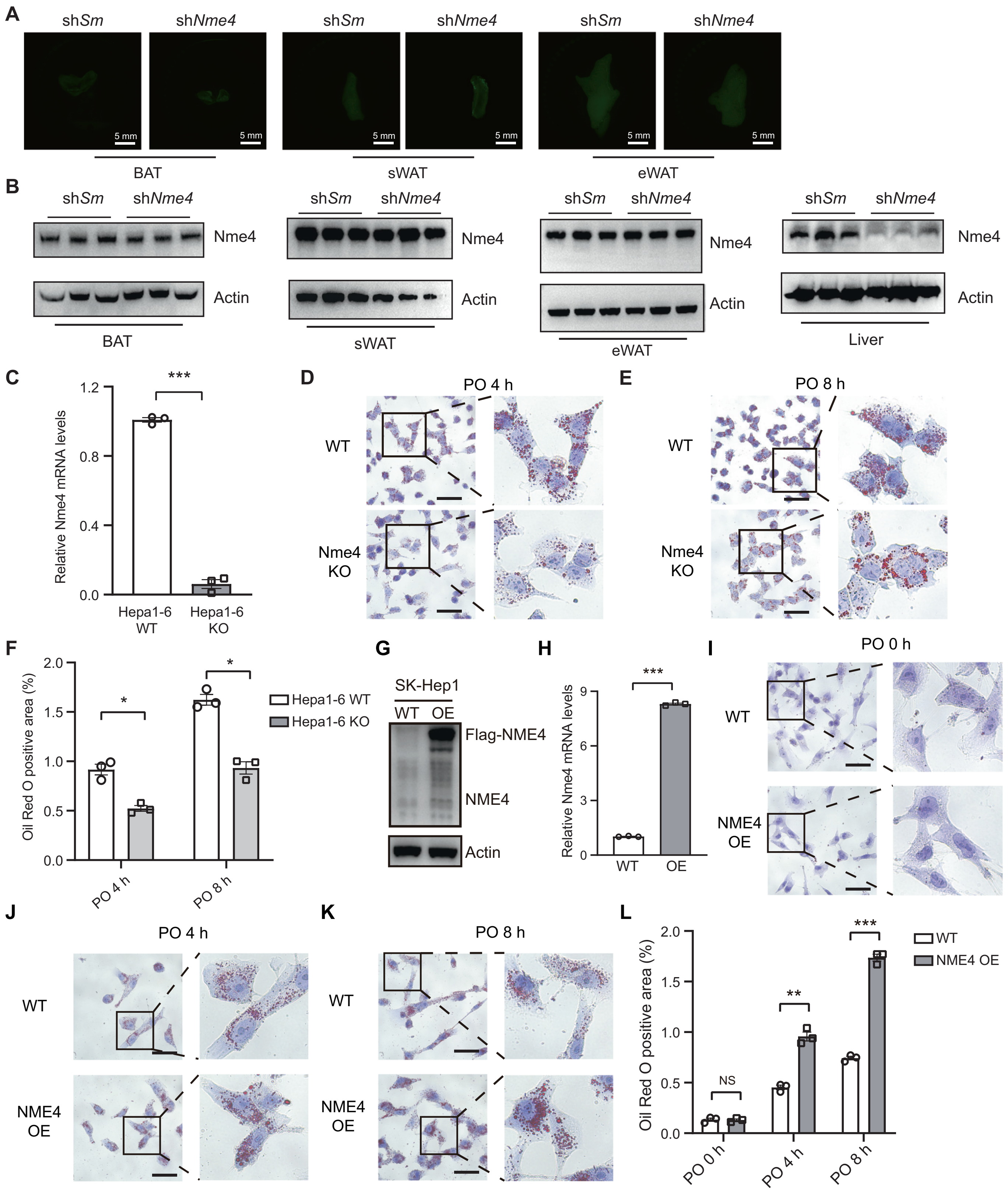
A
shSm
shNme4
shSm
shNme4
shSm
shNme4
5 mm
BAT
sWAT
eWAT
B
shSm
shNme4
Nme4
Actin
BAT
sWAT
eWAT
Liver
C
Relative Nme4 mRNA levels
***
Hepa1-6 WT
Hepa1-6 KO
D
PO 4 h
WT
Nme4 KO
E
PO 8 h
F
Oil Red O positive area (%)
Hepa1-6 WT
Hepa1-6 KO
PO 4 h
PO 8 h
G
SK-Hep1
WT OE
Flag-NME4
NME4
Actin
H
Relative Nme4 mRNA levels
WT
OE
I
PO 0 h
NME4 OE
J
K
L
WT
NME4 OE
NS
**
PO 0 h

© The Author(s)

Figure EV3. Depleting Nme4 in Hepa1-6 cells reduced lipid accumulation.

(**A**) The fluorescence imaging of BAT, sWAT and eWAT from mice with 3 weeks injection of AAV. Scale bars, 5 mm. (**B**) WB detect the Nme4 and Actin level in BAT, sWAT and eWAT from mice with 12 weeks injection of AAV. (**C**) The mRNA level of Nme4 in wild-type and Nme4-KO Hepa1-6 cells was measured by RT–qPCR. Biological replicates, $n = 3$. (**D**,**E**) Oil Red O staining of wild-type and Nme4-KO Hepa1-6 cells treated with PO for the indicated times. Scale bars, 50 μm. (**F**) The Oil Red O-positive areas in (**B**,**C**) were quantified by IPP. Biological replicates, $n = 3$. (**G**,**H**) The protein level (**E**) and mRNA level (**F**) of NME4 in wild-type and NME4-OE SK-Hep1 cells were measured by western blotting and RT–qPCR, respectively. Biological replicates, $n = 3$, unpaired Student's *t* test; ***P values < 0.001. Data are shown as the Mean ± SEM. (**I–K**) Oil Red O staining of wild-type and NME4-OE SK-Hep1 cells treated with PO for the indicated times. (**L**) The Oil Red O-positive areas in (**G–I**) were quantified by IPP. Biological replicates, $n = 3$. Data information: (**C**,**F**,**H**,**L**) data are presented as mean ± SEM. *P values ≤ 0.05, **P values ≤ 0.01, ***P values ≤ 0.001, NS -P values > 0.05 (Student's *t* test, unpaired). Sm scramble, sWAT subcutaneous white adipose tissue, eWAT epididymal white adipose tissue, BAT brown adipose tissue, KO knockout, OE overexpression, h hour.

 © The Author(s)

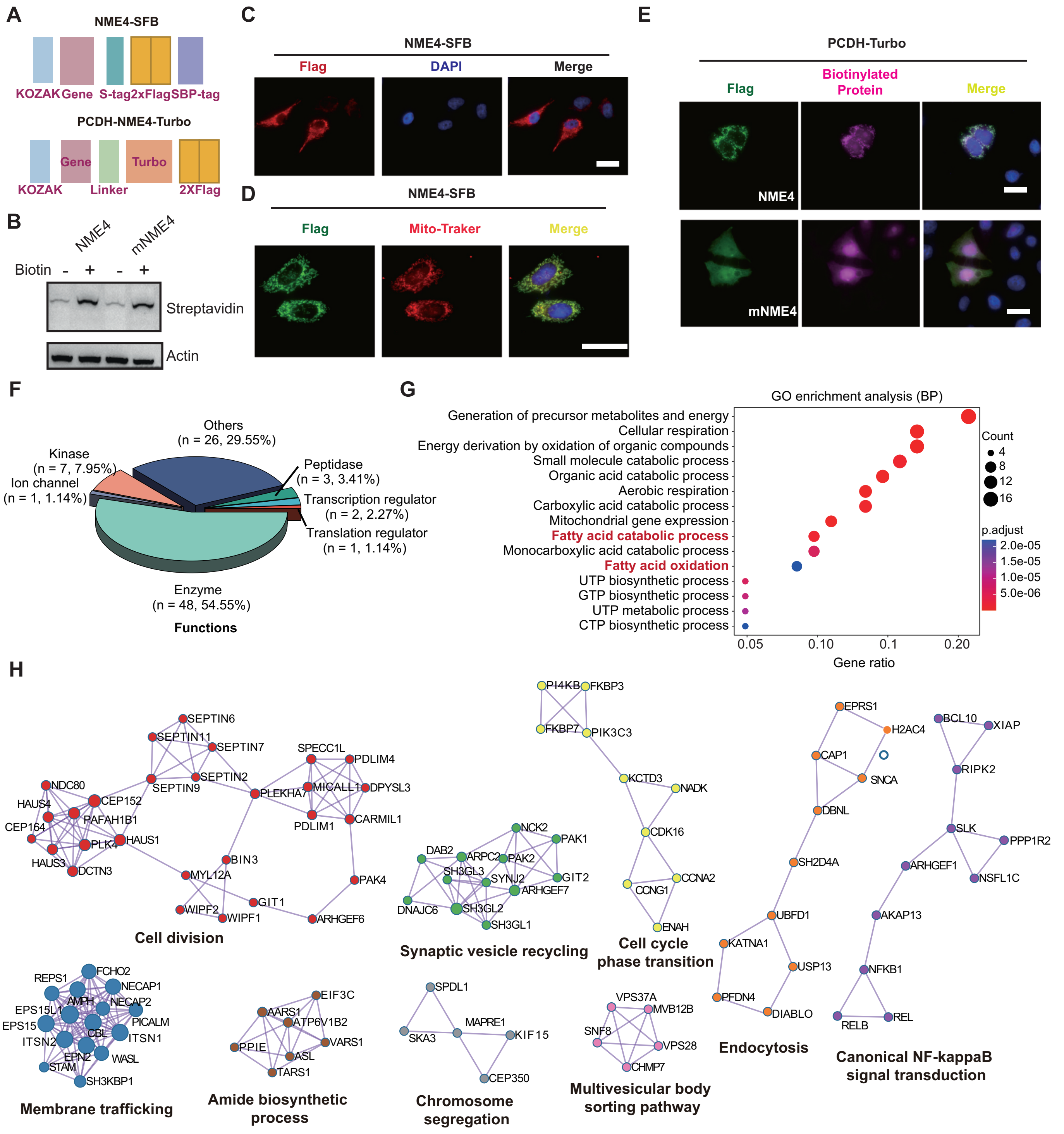

Figure EV4. Comprehensive interactome analysis revealed the composition and functions of NME4 interaction networks.

(**A**) Plasmid design for NME4 overexpression in TAP-MS and TurboID-MS. (**B**) HEK293T cells stably overexpressing Turbo-tagged NME4 and mNME4 were subjected to WB after treated with 500 μM biotin incubated in 37 °C for 30 min. Anti-biotinylated protein antibody is specifically recognized biotinylate protein, Actin as the internal control. mNME4, mitochondrial localization sequence-deleted in NME4. (**C–E**) HEK293T cells stably overexpressing SFB- or Turbo-tagged NME4 were subjected to immunofluorescence with an anti-Flag antibody to identify NME4, MitoTracker is to visualized mitochondrial, anti-biotinylated protein antibody and DAPI and visualized by microscopy. Scale bars, 50 μm. For biotinylated protein labeling, cells were treated with 500 μM biotin for 30 min. (**F,G**) Results of functional categories (**F**) and Biological Processes (**G**) enrichment analysis (GO) of significantly changed proteins in the TAP-MS HCIPs was analyzed in Hitplot. *P* values ≤ 0.05, here only showed the top 15 of Biological Processes enriched by upregulated proteins. (**H**) The pathways enriched in mNME4 HCIPs with related genes are shown analyzed in metascape database.

© The Author(s)

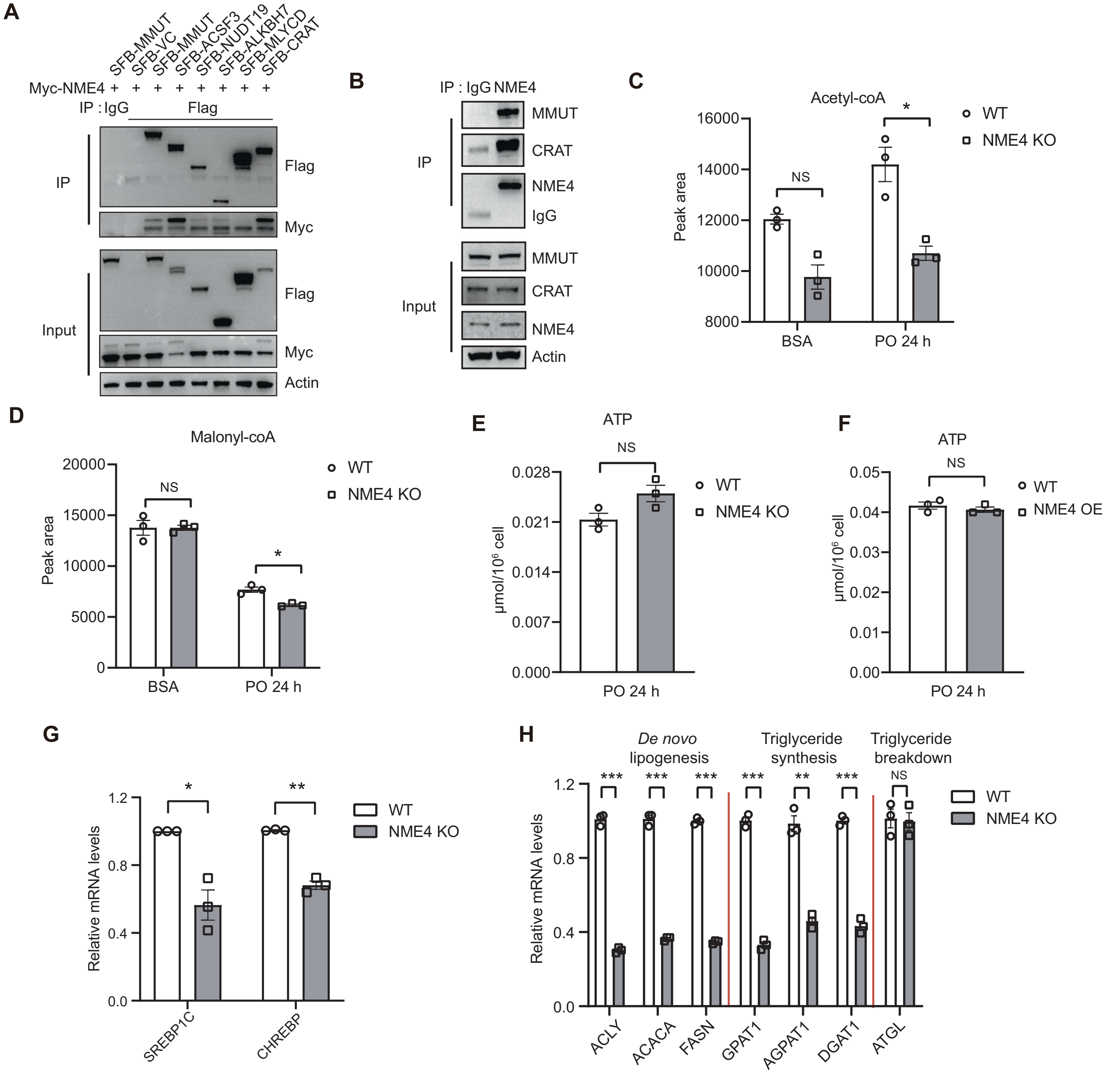

Figure EV5. NME4 regulates coenzyme A metabolism by interacting with the key enzymes in the pathway.

(**A**) HEK293T cells were cotransfected with Myc-tagged NME4 and C-terminal SFB (SFB)-tagged candidate genes, as indicated. The cell lysates were incubated with immunoglobulin G (IgG) control and antibodies against Flag. (**B**) HEK293T cell lysates were incubated with immunoglobulin G (IgG) control and antibodies against NME4. (**C**,**D**) Wild-type and NME4 KO Bel-7402 cells were treated with PO for 24 h. The acetyl-CoA (**C**) and malonyl-CoA (**D**) levels were determined by targeted metabolite analysis. Biological replicates, $n = 3$. (**E**) Wild-type and NME4 KO Bel-7402 cells were treated with PO for 24 h. ATP levels was measured by kit. Biological replicates, $n = 3$. (**F**) Wild-type and NME4-overexpression SK-Hep1 cells were treated with PO for 24 h. ATP levels was measured by kit. Biological replicates, $n = 3$. (**G**) Relative mRNA levels of SREBP1C and CHREBP were measured by RT–qPCR. Biological replicates, $n = 3$. (**H**) Relative mRNA levels of key genes involved in de novo lipogenesis, triglyceride synthesis and triglyceride breakdown were measured by RT–QPCR in wild-type and NME4 KO Bel-7402 cells. Biological replicates, $n = 3$. Data information: (**C–H**) data are presented as mean ± SEM. *P values ≤ 0.05, **P values ≤ 0.01, ***P values ≤ 0.001, NS -P values > 0.05 (Student's t test, unpaired).

 © The Author(s)
